# Effect of Customized Nutritious Breakfast and Nutrition Education on Nutritional Status of Preschool Children in Economically Underdeveloped Multi-Ethnic Areas: A Cluster Randomized Clinical Trial in Linxia, China

**DOI:** 10.3390/nu16142287

**Published:** 2024-07-16

**Authors:** Zhongquan Jiang, Chao Song, Mingxuan Shi, Runtong Chen, Ying Hong, Chong Zhang, Wenhao Zheng, Binshuo Hu, Liang Wang, Ying Zhang

**Affiliations:** 1 School of Public Health, Lanzhou University, 222 South Tianshui Road, Lanzhou 730000, China; 220220912411@lzu.edu.cn (Z.J.);; 2 Department of Developmental and Behavioral Pediatrics, The Children’s Hospital, Zhejiang University School of Medicine, National Clinical Research Centre for Child Health, Hangzhou 310051, China; 3 Gansu Provincial Maternity and Child-Care Hospital, Lanzhou 730050, China; 4 Department of Public Health, Robbins College of Health and Human Sciences, Baylor University, Waco, TX 76706, USA

**Keywords:** preschool students, nutritional intervention, economically underdeveloped multi-ethnic areas, cluster randomized clinical trial

## Abstract

The nutritional status of preschool children in economically underdeveloped multi-ethnic areas is a global concern. This study aimed to examine the effect of a 2.2-year cluster randomized clinical trial that provided customized nutritious breakfast and nutrition education to preschool children in Linxia County, China. A total of 578 children aged 3 to 6 years were enrolled. After the intervention, the incidence of undernourishment was significantly lower in the intervention group compared to the control group (8.73% vs. 9.92%, OR = 0.01 [95%CI 0.00, 0.39], *p* = 0.014). Additionally, children with non-Muslim dietary habits had a lower incidence of undernourishment compared to those with Muslim dietary habits (OR = 0.05 [95%CI 0.00, 0.88]; *p* = 0.010). The intervention group also had a lower prevalence rate of wasting (OR = 0.02 [95%CI 0.00, 0.40]; *p* = 0.011) and a higher mean BMI-for-age Z-score (*β* = 1.05 [95%CI 0.32, 1.77]; *p* = 0.005) compared to the control group. These findings suggest that providing nutritious breakfast and nutrition education is an effective strategy to improve the nutrition and health of preschool children, particularly in economically disadvantaged regions and among children with Muslim dietary habits.

## 1. Introduction

The issue of inadequate nutrition among children is increasingly severe in economically underdeveloped areas. Globally, the scale of this problem continues to expand, with statistics indicating that over 454 million children worldwide face undernourishment (e.g., stunting, underweight, and wasting) [1,2]. The preschool period (ages 3 to 6 years) demands significant quantities of various nutrients [3] and represents a crucial phase for the development of various abilities and indicators such as children’s growth, cognition, social skills, and psychological development [4]. Undernourishment among preschool children not only leads to temporary growth restriction, but also significantly impacts their future health, contributing to issues like reduced cognitive development and an increased risk of chronic illnesses [5,6].

The World Food Programme (WFP) has implemented a series of child nutrition improvement programs in underdeveloped regions globally [7,8,9]. In China, innovative pilot projects have been carried out across the provinces of Guangxi, Hunan, Sichuan, and Gansu, with a goal of improving the nutritional status of preschool children [10,11,12]. Among these projects, the preschool child nutrition improvement project in Gansu is a WFP pilot initiative in western China. The project in Gansu aims to enhance children’s nutrition and health by implementing diverse nutritional interventions, including the provision of free customized nutritious breakfasts, the enhancement of dining environments, recipe design, and health education for the promotion of eating habits. The findings of this study are derived from the preschool child nutrition improvement project conducted in Gansu.

Although nutritional improvement projects in economically underdeveloped regions have garnered widespread support, existing research has primarily focused on the impact of nutritional supplements on the growth and development of infants and young children (comprising components like proteins, vitamins (A, D, B1, B2, B12, and folic acid), and minerals such as calcium, iron, and zinc) [13,14,15,16,17]. The distribution of these nutritional supplements in economically underdeveloped areas heavily relies on local government support, thus potentially posing challenges in terms of the sustainability and accessibility of intervention measures. In contrast, in-school nutritious breakfast programs might contribute to reducing undernourishment [18] and were supported by previous studies observing an association between regular breakfast consumption and children’s healthy weight [19,20,21,22]. However, evidence regarding the relationship between breakfast utilization in underdeveloped areas and undernourishment in children remains inconclusive. Simultaneously, in economically underdeveloped areas, paid breakfast programs might deter preschool children from purchasing breakfast [23,24]. Hence, by focusing on macronutrient intake in preschool children and providing free nutritious breakfasts directly to preschool children in economically underdeveloped areas, we can effectively improve their dietary diversity [24] and ensure that they receive adequate nutrition. Moreover, through nutrition education, the tailored breakfast recipe could be more easily disseminated among the target population in the future.

During our previous study [24], it was discovered that in Linxia County, a region characterized by its diverse population comprising Dongxiang, Hui, and Han ethnic groups, there exist nutritional challenges such as insufficient dietary variety and inadequate nutrition among preschool children. Moreover, it is important to highlight that a substantial portion of the local population adopts a daily eating pattern of merely two meals a day, notably excluding breakfast. Furthermore, the dietary disparities between various ethnic groups, particularly Muslims, Han Chinese, and other ethnic communities [25], may also lead to different outcomes in future growth and development.

This study adopts a cluster randomized clinical trial design to evaluate the effect of the WFP preschool children’s nutrition improvement project on undernourishment among preschool children in economically underdeveloped multi-ethnic areas. The study hypothesizes that the targeted preschool children in schools where the nutritional improvement program is randomly implemented will exhibit lower rates of undernourishment after 2.2 years compared to the control group of preschool children [26]. This hypothesis is based on the intervention measures in the intervention group, including providing customized nutritious breakfast and nutrition education, which are expected to increase nutrient intake among preschool children, thereby improving their growth and development status.

## 2. Materials and Methods

### 2.1. Design and Sample

This study is a three-year cluster randomized clinical trial conducted in 17 public kindergartens in Linxia County, Gansu Province, China. The baseline participants were preschool children in the kindergarten’s junior class. Most kindergartens in Linxia County, including the 17 involved in this study, did not provide customized nutritious breakfast or scientific nutrition education. The 17 schools involved were randomly allocated to two groups: (1) intervention group including customized nutritious breakfast (see details below), and (2) control group without intervention measures such as nutritional recipes, breakfast, or nutrition knowledge training. This trial is registered at ClinicalTrials.gov (ChiCTR2200056916).

The primary outcome was the combined incidence rate of undernourishment (stunting, underweight, and wasting) among children who did not exhibit undernourishment at baseline. Secondary outcomes included the incidence and prevalence rates of stunting, underweight, wasting, and anemia, the prevalence rates of undernourishment, and changes in BMI-for-age Z-score (BMIZ).

This study obtained approvals from the Chinese Ministry of Agriculture and Rural Affairs, the WFP, the Gansu Provincial Department of Agriculture and Rural Affairs, and the Ethics Review Committee of the School of Public Health, Lanzhou University, following the relevant ethical standards. The study was approved by and conducted in accordance with the ethical standards of the Medical Ethics Committee School of Public Health, Lanzhou University. The full trial protocol is presented in Supplementary File S1. The recruitment of preschool children and written informed consents from parents and students were all conducted at the baseline survey (October 2020). At the endline follow-up, parents again signed the written informed consent for the endline survey. Neither parents nor students were compensated for their participation in the trial.

The WFP indicators considered in this study were the number of impoverished villages covered, the count of relatively impoverished and low-income populations, the percentage of left-behind children enrolled in kindergartens, infrastructure, willingness to participate, and the state of the supply chain. All the schools that met the following criteria were qualified to participate in this trial: (1) Relative Poverty Priority Principle: Kindergartens needed to cover impoverished villages in poor townships, with the proportion of impoverished children within the kindergarten not less than 60%. (2) Left-behind Children Priority Principle: The proportion of left-behind children in kindergartens should not be less than 60%. Either of these two conditions could be met to qualify. (3) Ethnic Minorities Priority Principle: Given Linxia County’s multi-ethnic residential setup, with a 40.13% Muslim population, priority was given to kindergartens in ethnic minority settlements focusing on impoverished and left-behind children. (4) Basic Meal Provision Conditions: Due to project-related constraints, kindergartens that had basic kitchen infrastructure or the potential to modify basic kitchen infrastructure were chosen for meal provision. (5) Local Willingness and Cooperation: This encompassed the willingness of rural governments, foundational conditions for nurturing or developing the supply chain, and the level of acceptance and cooperation of local villagers, particularly in pure ethnic minority settlement areas.

Out of a total of 150 kindergartens, 17 were chosen to participate in this project. The selected kindergartens were predominantly medium or small in size, with first-grade classes designed to accommodate 30–150 students. It should be noted that the number of students in the first grade of kindergartens varied across schools, ranging from 8 to 136. In September 2020, these 17 schools were randomly assigned to intervention and control groups using a random number generator. Ultimately, 8 kindergartens were included in the intervention group, while 9 kindergartens were included in the control group. All 17 schools willingly agreed to implement the intervention measures. The baseline survey was conducted in November 2020, and the research data were collected by trained researchers.

**Figure 1:**
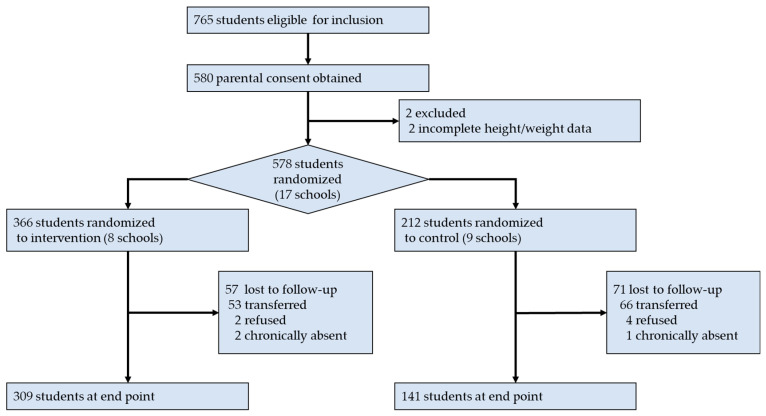
Consort diagram of student-level participation within 17 participating schools.

Intervention at schools began in March 2021 and lasted for three academic years (9 months/year of schooling). Final data were collected 2.2 years later, in May 2023. The CONSORT diagram was used to illustrate the process of the study at the child level (see Figure 1). Out of the 608 eligible students, 30 preschool children were excluded due to the following criteria: (1) the absence of parental or child consent (*n* = 5); and (2) substantial missing data (including missing demographic information, missing anthropometric data, or incomplete blood biomarkers, *n* = 25). Therefore, the total sample size for the baseline survey was 578 students.

During the study period, there were no reductions in the number of students at the schools. By the end of the study, 472 children (81.66%) remained. Approximately 71.70% of the study drop-outs were students who had transferred to another school. Supplementary Table S1 in the additional materials section demonstrates the sociodemographic characteristics of the students who remained and also those who were lost at the end of the study.

### 2.2. Study Intervention

#### 2.2.1. Intervention Schools

Eight intervention kindergartens had sample children (junior class in the 2020–2021 academic year, senior class in the 2022–2023 academic year) who participated and received the full intervention content. The following details describe the implementation of the intervention for the nutritional improvement pilot project for preschool children in Gansu’s Linxia County, which was jointly developed and provided by the Chinese Ministry of Agriculture and Rural Affairs, the WFP, the Gansu Provincial Department of Agriculture and Rural Affairs, and the School of Public Health at Lanzhou University.

#### 2.2.2. Nutritional Dietary Supplements

All preschool children in the sample kindergartens received a 2.2-year (9 months/year of schooling) customized nutritious breakfast. The breakfast recipe was designed by the School of Public Health, Lanzhou University, based on the dietary characteristics of the project area. It targeted providing macronutrients in proportions of 14% protein, 30% fat, and 56% carbohydrates, accounting for 30% of the total energy intake. This addressed issues of local micronutrient deficiencies and low dietary diversity, aiming to improve the nutritional status. The breakfast recipe for each grade is detailed in Supplementary File S2.

#### 2.2.3. Nutrition Education

During the trial period, sample children were exposed to educational animated videos on balanced diets and nutritional health, which were played continuously in the kindergartens. Additionally, there were at least three parent–child activities, garden tours, nutritional meal drawing contests, and supplementary micro-education courses for children.

#### 2.2.4. Social Marketing

Sample children and parents received various items printed with information on the project and brochures on balanced diets and nutritional health. Teachers and staff received posters for classrooms and dining areas.

#### 2.2.5. Knowledge Dissemination

All kindergarten directors, teachers, and staff participated in the biannual nutrition education training. After the training, they were required to disseminate the knowledge acquired to children and their guardians through parent meetings, home visits, etc.

#### 2.2.6. Control Schools

During the study period, control group schools did not undertake any targeted interventions in terms of nutrition education, social marketing, or knowledge dissemination to prevent undernourishment.

#### 2.2.7. Measures

Trained researchers conducted measurements on the children at baseline and after 2.2 years. The presence of evident nutrition education and social marketing in schools made it impossible for the researchers to employ blinding regarding whether a school belonged to the intervention group or the control group.

### 2.3. Measurements

#### 2.3.1. Height and Weight Measurements

Children’s height and weight were measured twice after a 10 h fast using a stadiometer (Model: SZ-200, Manufacturer: Suheng, Production: Suzhou, China) for height and an electronic health scale (Model: HD382, Manufacturer: Beurer, Production: Neu-Ulm, Germany) for weight. If the two measurements differed by more than 1.0 cm for height or 0.2 kg for weight, a third measurement was taken, and the average of the two closest measurements was used. The WHO Anthro (version 1.0.1) and WHO AnthroPlus software (version 0.9.0) were used to calculate Z-scores (height-for-age Z-scores (HAZ), weight-for-age Z-scores (WAZ), and weight-for-height Z-scores (WHZ), BMIZ) to evaluate stunting, underweight, and wasting for preschool children according to WHO guidelines [27].

Binary results were determined using the following thresholds:

- Stunting, underweight, and wasting: Under 5 years (stunting: HAZ < −2; underweight: WAZ < −2; wasting: WHZ < −2), 5 years and above (stunting: HAZ < −2; underweight: WAZ < −2; wasting: BMIZ < −2)
- Anemia: According to Gansu Maternal and Child Health Hospital standards (adjusted for altitude), HGB < 118 g/L (children under 5 years) and HGB < 123 g/L (children aged 5 and above).

#### 2.3.2. Hematology Measurements

All children had peripheral blood collected in EDTA vacutainer tubes before breakfast. Hemoglobin levels were analyzed using an automated blood analyzer (XS-500i, SYSMEX, Tokyo, Japan). Blood samples were stored and transported at 2–8 °C and tested within 12 h by professional technicians.

#### 2.3.3. Demographic Information

Children’s age, sex, ethnicity, and grade were obtained from the schools at baseline. The sample children’s ethnicity was primarily Dongxiang, Hui, and Han ethnic groups. The dietary habits were categorized based on the ethnic groups’ eating patterns as non-Muslim dietary habits and Muslim dietary habits.

### 2.4. Statistical Analysis

Preliminary data on incidence rates, retention rates, and design effects determined the statistical power for the primary study outcomes. It was estimated that the incidence rate of undernourishment in the intervention group would decrease by about 40% at the final measurement. The study assumed α = 0.05, a test power (1-*β*) of 80% or higher, an intraclass correlation coefficient (ICC) of 0.002 or lower, and an average of 35 junior class preschool children per school, to detect a risk ratio of 0.60 in a situation where the baseline incidence of undernourishment was 25% [13]. Therefore, a minimum of 16 schools was needed; see Supplementary Table S2. However, considering the limited number of students in some schools and the potential issue of sample attrition, we recruited 17 schools.

Continuous variables were presented as mean ± standard deviation (for normal distribution and homogeneity of variance) or median with interquartile range (IQR) for skewed distributions. Categorical variables were presented as percentages. The chi-squared (χ^2^) test or Fisher’s exact test compared ratios, and *t*-tests or Wilcoxon rank-sum tests compared means. The primary and secondary undernourishment outcomes were analyzed using an Independent Repeated Measures Generalized Estimating Equations (GEE) model to assess the intervention differences in the occurrence of outcomes at two measurements, excluding children positive for the current outcome at baseline. Two-sided *p* < 0.05 was considered statistically significant. Analysis was performed using R 4.2.1, with the implementation of GEE models using geepack (version 1.3.9), WHO Anthro using anthro (version 1.0.0), and WHO AnthroPlus using anthroplus (version 0.9.0).

## 3. Results

### 3.1. Participant Characteristics

The beginning of the study included 578 preschool children, with a median age of 3.57 ± 0.56. There were 321 males (55.54%). Of these, a total of 323 individuals (56.90%) had non-Muslim dietary habits. Statistically significant differences between the intervention and the control groups were observed in dietary habits (*p* < 0.05), as indicated in Table 1.

The undernutrition status for the participants at the beginning of the study was as follows: 39 individuals (6.75%) experienced stunting, 40 individuals (6.92%) were underweight, and 24 individuals (4.15%) had wasting, as indicated in Table 1. The differences in the prevalence of each undernourishment between the intervention and control groups were not significant (*p* > 0.05). At the end of the study, the numbers and prevalence rates for undernourishment indicators of the remaining 450 study participants were as follows: 4 individuals (0.89%) experienced stunting, 19 individuals (4.22%) were underweight, and 54 individuals (12.00%) had wasting, as indicated in Table 2.

### 3.2. Nutritional Status

At the end of the study 2.2 years later (9 months/year of schooling), the intervention schools (8.73% [24 students out of 275]) and control schools (9.92% [12 students out of 121]) showed a significant difference in the primary outcome’s malnutrition incidence rate. The intervention notably reduced the incidence rate of undernourishment among the sampled children (OR = 0.01 [95%CI 0.00, 0.39]; *p* = 0.014). Simultaneously, the incidence rate of undernourishment among non-Muslim dietary habits significantly decreased compared to children with Muslim dietary habits (OR = 0.05 [95%CI 0.00, 0.88]; *p* = 0.010). There was also a statistically significant interaction between the intervention measures and dietary habits in the incidence rate of undernourishment (*p* = 0.018). Pure stunting, underweight, and wasting incidence rates showed no significant differences between the intervention and control groups; see Table 3.

For other secondary prevalence outcomes, the intervention schools showed a significantly lower prevalence rate of wasting compared to the control schools (OR = 0.02 [95%CI 0.00, 0.40]; *p* = 0.011), as shown in Table 2. In addition, the undernourishment prevalence rate among intervention schools significantly decreased compared to control schools (OR = 0.04 [95%CI 0.00, 0.65]; *p* = 0.024). Other prevalence rates related to stunting, underweight, and anemia showed no significant differences between the intervention and control groups.

The intervention shows a significant association with the BIMZ. The mean BIMZ in the intervention group is significantly higher than that in the control group (*β* = 1.05 [95%CI 0.32, 1.77]; *p* = 0.005). Simultaneously, there is a significant interaction between the intervention measures and dietary habits concerning the BIMZ (*β* = −0.56 [95%CI −1.01, −0.11]; *p* = 0.016).

## 4. Discussion

The results of this study showed that the provision of customized nutritious breakfast and nutrition education intervention significantly reduced the incidence of undernutrition among preschool children in underdeveloped multi-ethnic regions (OR = 0.01 [95%CI 0.00, 0.39]; *p* = 0.014). Nutritional intervention had a positive impact on children’s growth and development. Other studies targeting school-age children have indicated that nutritional intervention measures can improve children’s nutritional knowledge and change their food preferences [28,29,30,31], thereby improving the growth and development status of preschool children through these pathways. The incidence and prevalence of undernutrition decreased, and the prevalence of wasting also showed a decrease. This finding suggests that wasting preschool children exhibited a positive trend in growth and development after receiving additional energy intake through the provision of customized nutritious breakfast and nutrition education. This finding is consistent with previous research evidence and supports the hypothesis of this study [26,32,33,34]. Previous studies [26,32,33,34] have consistently shown an association between regular breakfast consumption in children and a decreased risk of stunting, underweight, and wasting. It is worth noting that non-Muslim diets in economically underdeveloped regions may be a protective factor against undernutrition compared to Muslim dietary habits, possibly due to the fact that Muslims primarily consume beef and mutton as their main source of red meat [25]. However, beef and mutton are often more expensive [35]. Therefore, in economically underdeveloped areas where price sensitivity is a significant factor, the adoption of the Muslim dietary habits may lead to a partial decrease in the consumption of red meat [36]. Consequently, the adherence to Muslim dietary habits among preschool children may contribute to a higher incidence of undernutrition in economically underdeveloped areas. In future nutritional interventions, it is essential to tailor the supplementation of fat and protein intake according to dietary habits. Meanwhile, it is important to pay attention to the adequacy of macronutrient intake in the nutritional supplementation of preschool children in underdeveloped areas with Muslim dietary habits. Subsequently, targeted measures such as health education or dietary supplementation should be implemented to improve the nutritional status of children.

During the intervention process, an intriguing observation emerged: as the rates of stunting and underweight decreased, there was a noticeable rise in the prevalence of wasting among the children. This phenomenon can be explained by the lack of synergy in the development of children’s height and weight observed in previous studies [37,38]. It is widely recognized that preschoolers undergo a period of rapid height growth, while weight gain progresses at a comparatively slower pace [37,38]. This discrepancy may be attributed to the structural changes in their bodies and the ongoing skeletal growth, which outpaces the growth of fat and muscle. Notably, the evaluation indicator BMIZ, used to assess the prevalence of wasting, demonstrates a significantly larger increase in the quadratic term of height (Height^2^) compared to the linear term of weight. Consequently, this leads to an overall escalation in the incidence and prevalence of wasting.

Taking into consideration the inherent influence of dietary habits among the Muslim groups [25], the interaction between the intervention measures and dietary habits was examined in the GEE analysis. The inclusion of interaction terms increased the complexity of the GEE model [39], resulting in an increased uncertainty of the estimates and wider confidence intervals. The width of the confidence intervals does not necessarily imply a lack of significance in the relationship between variables, but rather reflects the uncertainty of the estimates.

This study has several limitations. First, the findings might not be generalizable to other urban areas or regions with different demographic profiles. Second, to comply with ethical standards, the choice of breakfast for children in the control group was not restricted. This might have resulted in inconsistent dietary habits and nutritional intake. This inconsistency likely impacts the results, making it challenging to distinguish if the effects in the intervention group were due to the intervention or other factors. Third, a significant portion (92.97%) of the sample loss was due to children transferring to other schools. However, it was unrelated to the characteristics of the preschool-aged children, which greatly reduced the selection bias. Finally, although there was no significant reduction in the number of schools that may avoid a decrease in statistical power, the study might not have achieved the expected statistical power concerning the detected effect size.

## 5. Conclusions

The provision of the customized nutritious breakfast and nutrition education was observed to have a positive effect on the nutritional status of preschool children in economically underdeveloped areas. Specifically, the nutritious breakfast had a significantly positive effect on the BIMZ of children with Muslim dietary habits. These findings underscore the significance of providing nutritious breakfast and nutrition education in economically disadvantaged regions, particularly in improving the nutrition and health of Muslim children.

## Figures and Tables

**Table 1:** Baseline characteristics of the 578 preschoolers participating in the project.

Category	Total	Intervention Group	Control Group	*p* Value
	*N* = 578	*n* = 366	*n* = 212	
BMI	14.50 ± 1.15	14.55 ± 1.24	14.43 ± 0.97	0.221
BMIZ	0.86 ± 0.83	0.90 ± 0.89	0.89 ± 0.71	0.283
Age	3.57 ± 0.56	3.54 ± 0.56	3.61 ± 0.59	0.163
Gender				0.698
Male	321 (55.54%)	206 (56.28%)	115 (54.25%)	
Female	257 (44.46%)	160 (43.72%)	97 (45.75%)	
Dietary habits				0.006
non-Muslim	323 (56.90%)	244 (62.50%)	98 (46.20%)	
Muslim	255 (44.10%)	122 (38.50%)	114 (53.80%)	
Undernutrition status				
Stunting	39 (6.75%)	23 (6.28%)	16 (7.55%)	0.681
Underweight	40 (6.92%)	24 (6.56%)	16 (7.55%)	0.870
Wasting	24 (4.15%)	15 (4.10%)	9 (4.25%)	1.000

Abbreviations: BMI: Body Mass Index; BMIZ: BMI-for-age Z-scores.

**Table 2:** Nutritious breakfast intervention trial participants’ undernutrition prevalence status at endpoints.

Outcome		Baseline	Endpoint	Intervention	*p* Value	Dietary Habits (Non-Muslim and Muslim)	*p* Value	Intervention * Dietary Habits Interaction Terms	*p* Value
				Odds Ratio (95%CI)		Odds Ratio (95%CI)		Odds Ratio (95%CI)	
Secondary Outcomes									
	Prevalence of stunting			-	-	-	-	-	-
	Intervention group	23/366	1/309						
	Control group	16/212	3/141						
	Prevalence of underweight			1.57 (0.00, 113.42)	0.907	14.99 (0.14, 129.15)	0.236	0.67 (0.05, 9.27)	0.764
	Intervention group	24/366	9/309						
	Control group	16/212	10/141						
	Prevalence of wasting			0.02 (0.00, 0.40)	0.011	0.12 (0.01, 1.02)	0.052	4.93 (1.43, 16.97)	0.011
	Intervention group	15/366	36/309						
	Control group	9/212	18/141						
	Prevalence of undernourishment			0.04 (0.00, 0.65)	0.024	0.30 (0.05, 1.97)	0.209	3.30 (1.07, 10.14)	0.037
	Intervention group	43/366	38/309						
	Control group	27/212	24/141						
	Prevalence of anemia			3.20 (0.00, 27.25)	0.756	2.64 (0.02, 34.06)	0.705	0.75 (0.05, 11.87)	0.835
	Intervention group	6/309	6/304						
	Control group	2/141	2/140						

Intervention * Dietary Habits Interaction Terms: interaction effects between intervention and dietary habits.

**Table 3:** Nutritious breakfast intervention trial participants’ undernutrition incidence status and BMIZ results at endpoints.

Outcome		Baseline	Endpoint	Intervention	*p* Value	Dietary Habits (Non-Muslim and Muslim)	*p* Value	Intervention * Dietary Habits Interaction Terms	*p* Value
				Odds Ratio (95%CI)		Odds Ratio (95%CI)		Odds Ratio (95%CI)	
Primary Outcomes									
	Incidence of undernourishment			0.01 (0.00, 0.39)	0.014	0.05 (0.00, 0.88)	0.010	6.92 (1.39, 34.29)	0.018
	Intervention group	-	24/275						
	Control group	-	12/121						
Secondary Outcomes									
	Incidence of stunting			-	-	-	-	-	-
	Intervention group	-	0/291						
	Control group	-	1/129						
	Incidence of underweight			0.65 (0.09, 25.26)	0.094	3.34 (0.12, 111.43)	0.628	1.06 (0.06, 18.45)	0.969
	Intervention group	-	6/292						
	Control group	-	4/128						
	Incidence of wasting			0.03 (0.00, 1.03)	0.052	0.18 (0.020, 1.94)	0.158	3.87 (0.99, 15.20)	0.053
	Intervention group	-	28/295						
	Control group	-	12/134						
	Incidence of anemia			3.20 (0.00, 28.41)	0.756	2.64 (0.02, 34.06)	0.705	0.75 (0.05, 11.87)	0.835
	Intervention group	-	6/304						
	Control group	-	2/140						
				*β*		*β*		*β*	
	BMIZ			1.05 (0.32, 1.77)	0.005	0.18 (−0.17, 0.52)	0.322	−0.56 (−1.01, −0.11)	0.016
	Intervention group	0.90 ± 0.89	−0.69 ± 1.37						
	Control group	0.89 ± 0.71	−0.93 ± 1.05						

Intervention * Dietary Habits Interaction Terms: interaction effects between intervention and dietary habits.

## Data Availability

The data presented in this study are available on request from the corresponding author. The data are not publicly available due to confidentiality issues.

## Supplementary Materials

The following supporting information can be downloaded at: https://www.mdpi.com/article/10.3390/nu16142287/s1, Supplementary File S1: The full trial protocol. References [1,2,8,9,10,11,24] are cited in the supplementary materials; Supplementary Table S1: Demographic differences between drop-out and retention samples; Supplementary Table S2. Tests for two proportions in a cluster-randomized design; Supplementary File S2: The breakfast recipe for each grade.
