# Supplementary material for: Effect of Customized Nutritious Breakfast and Nutrition Education on Nutritional Status of Preschool Children in Economically Underdeveloped Multi-Ethnic Areas: A Cluster Randomized Clinical Trial in Linxia, China
Source: Nutrients. 2024 Jul 16;16(14):2287. doi: 10.3390/nu16142287 (PMC11280342; doi:10.3390/nu16142287)
Supplement: Supplementary file 1 [file nutrients-16-02287-s001.zip › Supplementary File S1.pdf]

THE FULL TRIAL PROTOCOL

1. STUDY TITLE

The United Nations World Food Programme Gansu Pilot Project on Nutrition Improvement for Preschool Children (WFP-GPNP)

2. OBJECTIVES

The main research question in Phase I is to evaluate the feasibility and acceptability of implementing the "World Food Programme Gansu Pilot Project on Nutrition Improvement for Preschool Children(WFP-GPNP)" in 17 kindergarten classes during the 2020-2021 academic year. Specific objectives include:

1. Based on the background of the "China Food and Nutrition Development Program (2014-2020)", "National Nutrition Plan (2017-2030)", and the WFP-GPNP, develop school nutrition intervention measures, including free breakfast design and nutrition education programs targeting health issues in the project area.
2. Conduct a pilot feasibility study in 8 intervention schools and 9 control schools to evaluate feasibility and acceptability.

The main research question in Phase II is to evaluate the impact of the WFP-GPNP on the overall incidence of malnutrition (stunting, underweight, and wasting) among preschool children in 17 kindergartens from the 2021-2022 academic year to the 2022-2023 academic year. Specific objectives include:

1. Implement free breakfast and nutrition education programs targeting health issues in the project area in 8 intervention kindergartens under the background of the WFP-GPNP.
2. Conduct a randomized controlled trial (RCT) in 17 schools (8 intervention schools and 9 control schools) to compare the incidence of overnutrition (stunting, underweight, and wasting) in the two groups.

3. BACKGROUND

BACKGROUND AND SIGNIFICANCE

The need for prevention. Child malnutrition is a global problem, especially in economically underdeveloped rural areas, which affects children's growth and future health [1, 2]. The United Nations World Food Programme (WFP) has implemented a project to improve the nutrition of pre-school children in China, aiming to improve children's dietary diversity and nutrition status by providing free breakfast and nutrition education [3-5]. This study uses a cluster randomized clinical trial to evaluate the effectiveness and influencing factors of this project. The study hypothesizes that this project can effectively reduce child malnutrition and improve children's health levels.

RATIONALE

Growth and Development Issues in Underdeveloped Areas. Baseline surveys have revealed that the preschool children in the area have poor growth and development levels (with a stunting rate of 6.61%, low birth weight rate of 7.11%, and malnutrition rate of 3.97%). Additionally, their dietary intake is inadequate (with only 71.40% meeting the average energy and macronutrient requirements), and they lack essential micronutrients (with a deficiency rate of 29.35% for calcium, iron, and zinc). Furthermore, there is a high prevalence of dental caries (54.20%) and a significant number of children skipping breakfast.

Childhood Dietary Behavior Issues. The detection rate of childhood dietary behavior problems is 68.3%, which is relatively high compared to the national average. The highest proportion is found in children with six or more diet-related issues, accounting for 24.1%. It has been observed that the

occurrence of childhood dietary behavior problems is associated with factors such as age, birth weight, place of residence, parents' out-migration for work, and family size.

Furthermore, the survey revealed parental feeding behaviors, with permissive feeding scoring the highest average value (3.59 ± 0.73) on a scale of 5. This indicates it is the most common feeding behavior exhibited by parents. Other feeding methods ranked from highest to lowest include "prompting to eat" (3.36 ± 0.78), "encouraging to eat" (3.19 ± 0.69), and "forcing to eat" (2.83 ± 0.82). These scores suggest that inappropriate feeding methods are prevalent among the parents of the sampled children. Additionally, caregivers, kindergarten teachers, principals, and chefs all exhibited low nutritional literacy. These findings provide crucial information regarding the growth and development of children, breakfast consumption, and caregivers in rural underdeveloped areas. We will utilize this data to effectively develop intervention measures for our project.

4. ELIGIBILITY CRITERIA

In Phase I, participants will consist of junior class students from 17 kindergartens. In Phase II, participants will be followed from when they are junior class students in the 17 kindergartens until they transition to senior class students. The 17 schools included in the study were randomly assigned to one of the following two possibilities: 1. Intervention measures including providing free nutritious breakfast. 2. No intervention measures provided, including nutrition recipes, nutritious breakfast, and nutrition knowledge training. The primary outcome is the composite incidence of undernutrition (stunting, low birth weight, and malnutrition) among students who did not have undernutrition at baseline (proportion of new cases of undernutrition). Based on our previous research, no schools declined the project intervention measures.

5. TREATMENT PLAN

Phase I

Study population: In Phase I, out of 150 schools, 17 kindergartens were selected based on criteria such as relative poverty priority, left-behind children priority, ethnic minority priority, meal provision infrastructure, and local willingness and collaboration. The participants are junior class students from these 17 kindergartens in Linxia County, Gansu Province. Based on the total enrollment of each school, we estimate a total of 580-650 eligible students for Phase I, with an average of 35 junior class students per school.

Phase I includes determining school eligibility, selecting schools for intervention implementation, conducting baseline surveys in November 2020 to prepare for the development of nutritious breakfast recipes and nutrition education, and hosting expert consensus meetings to assess the feasibility and acceptability of the intervention methods.

Consent forms/authorization letters will be collected in October 2020. At the same time, baseline measurement data will be collected in all 17 schools, including height and weight, demographic information (age, grade, date of birth, gender, and ethnicity), blood samples, and dental caries examination.

After completing the baseline measurements, a 2-week free nutritious breakfast menu will be designed for a three-year period based on the baseline growth and development status of children in the project area. Additionally, nutrition education courses will be designed for children, caregivers, teachers, principals, and chefs.

Phase I will also include a parental component. The parental component of the WFP-GPNP study will involve parents filling out a survey questionnaire (dietary survey, child dietary behavior survey, feeding practices survey, caregiver nutrition knowledge, attitudes, and behavior survey). Based on the

total number of students in the pilot study, assuming approximately 95% of registered students agree to participate in Phase I of the study, and 90% of the participating students' parents fill out the survey questionnaire with the help of interviewers, the estimated total sample size for the parental component in Phase I is 496-555 individuals. The purpose of Phase I is to assess the feasibility and acceptability of the SBPI. Based on the data obtained from this baseline survey, intervention measures and research materials will be improved for future comprehensive implementation.

Phase II

Study Population. In Phase II, participants will be continuously tracked from the qualified sample of children identified in the baseline survey of Phase I. These children are the junior class students from the 17 kindergartens, and they will be followed until they transition to senior class. The estimated total number of qualified samples in Phase I is 580-650 students, assuming an 80% loss to follow-up rate for the sample children, the final sample size would range from 464-520.

Phase II will involve a three-year RCT study conducted in 8 intervention schools and 9 control schools to evaluate the impact of customized free nutritious breakfast and nutrition education on the composite incidence of undernutrition (stunting, low birth weight, and malnutrition) among children. Consent forms/authorization letters will be collected in May of the 2023-2024 school year. In May 2013, final measurement data will be collected in all 17 schools, including height and weight, demographic information (age, grade, date of birth, gender, and ethnicity), blood samples, and dental caries examination.

The free customized nutritious breakfast will cover all children in the 8 kindergartens (approximately 1600 individuals), including about 300 junior class children. Nutrition education will be conducted for 3 years, including no less than 6 sessions of nutrition and health training, no less than 6 sessions of social and behavioral knowledge dissemination, and no less than 6 sessions of psychological counseling for left-behind children. It will cover approximately 400 parents and 80 staff members.

Phase II will also include a parental component. The parental component of the WFP-GPNP study will involve parents filling out a survey questionnaire (dietary survey, child dietary behavior survey, feeding practices survey, caregiver nutrition knowledge, attitudes, and behavior survey). The estimated total sample size for the parental component in Phase II is 470 individuals. Once the collected data is entered and cleaned, preliminary analysis will begin in October 2023.

RANDOMIZATION

Schools will be randomly assigned to either the control group or the intervention group. A computer-generated numbering system will be used for random allocation to ensure that there is no bias in the random assignment.

Intervention Arm. WFP-GPNP aims to improve the growth and development status of preschool children in underdeveloped areas. In Phase I, the survey project primarily focuses on the growth and development issues of preschool children, and based on the identified problems, customized nutritious breakfast and nutrition education programs are developed. Expert meetings are held for multi-party discussions to assess the feasibility and acceptability of the interventions. In Phase II, the 8 intervention schools will receive intervention measures, including the following four strategies, to improve breakfast participation rates. These intervention measures will be adjusted based on feedback from principals and schools.

1. **Nutritional Dietary Supplements**. All preschool children in the sample schools will receive free nutritious breakfast for a duration of 2.2 years. The breakfast menus are designed by the School of Public Health at Lanzhou University, taking into account the dietary characteristics

of the project area. The proportions of macronutrient supply for children are as follows: protein 14%, fat 30%, carbohydrates 56%, and breakfast should provide 30% of total energy intake. The menus primarily address issues such as local micronutrient deficiencies and low dietary diversity leading to poor nutritional status. The two-week breakfast menus for each grade can be found in **Supplementary** 2.

2. **Nutrition Education**. During the trial period, sample children will receive educational animated videos on balanced diet and nutrition health, which will be continuously played in the kindergartens. Additionally, at least three parent-child activities, garden tours, nutrition meal painting competitions, and supplementary micro-lessons will be conducted for the children. These activities and lessons serve as supplements to the regular nutrition education for children.
3. **Social Marketing**. Sample children and their parents will receive various items and brochures promoting balanced diet and nutrition health. Teachers and staff members will receive promotional posters for classrooms and dining areas.
4. **Knowledge Dissemination**. All principals, teachers, and staff members of the kindergartens will participate in nutrition education training activities twice a year. After the training, the kindergarten principals, teachers, and staff members are required to disseminate the knowledge they have learned to the children and their guardians through parent meetings, home visits, and other means.

Control Arm. The control group schools will only undergo data collection at baseline and endline, including height/weight measurements, demographic information, and questionnaire data. No placebo intervention will be provided. There will be no intervention on breakfast consumption in the control group.

CONSENT

Students and Parents. Prior to conducting any research procedures, parental and student consent must be obtained. Parental consent and student assent include allowing the research team to collect each child's height and weight, demographic information (including age, gender, date of birth, grade, and ethnicity), questionnaire survey data, and blood samples. The consent forms also include permission for conducting breakfast interventions for the sample children in the intervention group.

The parental consent form/student assent form will be signed twice, at baseline and endline, respectively. Researchers will coordinate with teachers to hold two 15-minute information sessions to explain the study details, consent forms, and collect completed forms. The sessions will emphasize that participation in the study is entirely voluntary and there will be no punishment for non-participation. Each student and one parent or guardian must sign the forms. In Phase I, it will also be explained that the baseline data collected will be used for the design of the nutritious breakfast and nutrition education, and that only 8 out of the total 17 schools will receive the intervention, while the other schools will only participate in measurements (height, weight, breakfast questionnaire, and demographic data). Additionally, it will be explained that all students in the intervention schools will participate in the same activities (free nutritious breakfast, nutrition education, etc.), regardless of their consent/assent. Finally, it will be clarified that if a student or parent/guardian changes their mind and no longer wishes to participate after submitting a completed consent form, they can withdraw their consent at any time.

Principals. For the parental component, questionnaire surveys will be conducted at the schools in October 2020 during Phase I and in May 2023 during Phase II. The research team will organize a

survey team to visit the schools and conduct the questionnaire surveys. Parents who complete the caregiver questionnaire section (demographic survey, dietary survey, sample child's eating behavior survey, feeding practices survey, caregiver's nutrition knowledge, attitudes, and behavior survey) will receive a gift worth ¥15 as a token of appreciation for their participation.

Principals. The school principals will provide consent for their schools to participate in either the intervention or control group of the study. In Phase I, after the completion of the baseline survey, schools will be randomly assigned to the intervention or control group. In Phase II, if a school is randomly assigned to the intervention group, all children in the nursery, middle, and upper classes of the intervention group kindergarten will receive the free nutritious breakfast intervention and nutrition education. The principals will also provide the research data for the children and their caregivers to the researchers.

Confidentiality. Parental consent and student assent will be obtained to execute all outlined research procedures, including allowing the collection of demographic information (gender, grade, age, date of birth, and ethnicity), height and weight measurements, and questionnaire data for each child. Additionally, permission will be sought to collect dining data. Parental consent and student assent will also be obtained to execute all expert discussion procedures outlined. Additional measures will be taken to ensure confidentiality for the students. Measures will be taken in a private space while the students are dressed. During the expert discussion process, no personally identifiable information, including the names of the students and parents, will be used or mentioned. All staff members will receive training. All child records will be stored as securely and confidentially as possible and labeled with ID numbers instead of the children's names. Only in a separate log will the children's names be linked to the ID numbers. Hard copies of the data will be kept in a locked drawer controlled by the coordinator at Lanzhou University. Completed questionnaires will be stored in a locked file.

METHODS

Height and weight measurements. Children's height and weight will be measured twice after a 10-hour fasting period. Height will be measured using a stadiometer (SZ-200, Suheng), and weight will be measured using an electronic health scale (HD382, Beurer). If the height and weight measurements differ by more than 1.0 cm and 0.2 kg, respectively, a third measurement will be taken, and the average of the two measurements within the specified range will be calculated. Based on the WHO-recommended growth indicators for preschool children, WHO Anthro and WHO AnthroPlus will be used to calculate z-scores for children under 5 years old and children 5 years and above, respectively, for height-for-age z-score (HAZ), weight-for-age z-score (WAZ), weight-for-height z-score (WHZ), and BMI-for-age z-score (BMIZ) [6]. These z-scores will be used to assess the prevalence of stunting, underweight, and wasting among the sample children.

The following cutoff values will be used to determine binary outcomes:

Stunting, wasting, and underweight: Under 5 years old (HAZ < -2, WHZ < -2, WAZ < -2), 5 years and above (HAZ < -2, WHZ < -2, BMIZ < -2)

Hematology Measurements. All children had peripheral blood collected in EDTA vacutainer tubes before breakfast. Hemoglobin levels were analyzed using an automated blood analyzer (XS-500i, SYSMEX, Tokyo, Japan). Blood samples were stored and transported at 2-8°C and tested within 12 hours by professional technicians.

Anemia: According to the standards of the Gansu Maternal and Child Health Hospital (adjusted for altitude), HGB < 118 g/L (children under 5 years old) and HGB < 123 g/L (children 5 years and above).

Demographic information: Age, gender, ethnicity, and grade of the preschool children will be obtained from the school at baseline. Based on the baseline results, the majority of the sample children belong to the Han, Hui, and Dongxiang ethnic groups.

Parental questionnaire. The caregiver questionnaire includes demographic survey (gender, age, ethnicity, current residence, education level, household size), dietary survey, sample child's eating behavior survey, feeding practices survey, and caregiver's nutrition knowledge, attitudes, and behavior survey.

6. RISKS

This study poses minimal risks to the participants. Participants, individuals, or school districts are not required to pay any fees. All measurements will be conducted during non-core curriculum time. To minimize discomfort and protect privacy, human measurements (height and weight) and demographic information will be collected and recorded in a safe and private environment. Students do not need to undress. For all self-administered questionnaires and surveys, there will be a distance between students and parents, and only the research identification number will be recorded. The forms will be collected directly by the researchers.

7. BENEFITS

The participants in this study do not directly benefit from their participation; however, the data collected from this study may contribute to research on intervention measures to address child malnutrition in economically disadvantaged rural kindergarten settings in the future. The intervention measures of this study could potentially influence future government interventions for addressing child malnutrition in underdeveloped areas, thereby bringing potential health benefits to parents and children. There are also potential benefits to science and public health, as the results of this study may make important and substantive contributions to the understanding of how to promote breakfast in schools in the education and health sectors.

In Phase I, the schools and school districts participating in the study will benefit from the height and weight measurements conducted by researchers from Lanzhou University. In October 2020, researchers from Lanzhou University will measure the height and weight of all kindergarten children in the participating 17 schools (580-650). In Phase II, the participating schools will benefit from the height and weight measurements of all preschool children in May 2023, conducted by researchers from Lanzhou University. The researchers will provide the data to the school doctors for reporting to the government. In Phase II, all the intervention group schools, covering a total of 1600 children, will receive free nutritious breakfasts.

8. ALTERNATIVE TREATMENT

The alternative is to not participate

9. DATA COLLECTION AND STATISTICS

Analysis. Continuous variables will be presented as means ± standard deviations (for normally distributed and homoscedastic data) or as medians with interquartile ranges (IQR) (for skewed data). Categorical variables will be presented as percentages. Chi-square test or Fisher's exact test will be used to compare proportions, while t-test and Wilcoxon rank-sum test will be used to compare means. The analysis of primary malnutrition outcomes and secondary outcomes will be conducted using an Independent Repeated Measures GEE (Generalized Estimating Equations) model to assess the intervention differences in the occurrence rates of the outcomes between the two measurements. Children with positive baseline outcomes for each outcome will be excluded from the occurrence rate model. A two-sided $P<0.05$ will be considered statistically significant. The analysis will be performed

using R 4.2.1, with the implementation of the GEE model using geepack (version 1.3.9), the implementation of WHO Anthro using anthro (version 1.0.0), and the implementation of WHO AnthroPlus using anthroplus (version 0.9.0).

Intervention Effects. Schools are potential sources of dependence; participants within the same school (i.e., cluster) often have common selection factors or exposures, making the observed outcomes of participants from the same cluster likely to be correlated. To account for potential correlation at the school level and differences between schools, we will include school as a fixed-effect variable in the analysis of covariance (ANCOVA) and use the baseline measurement as a covariate. With this variance analysis design, we will test the effect of the intervention compared to the control condition on weight change (pre-post).

The outcome of interest is the composite incidence rate of malnutrition (stunting, low weight, and wasting) (proportion of new cases of malnutrition among students who did not have malnutrition at baseline). The statistical power for the primary study outcome was determined based on previous research, incidence rates, retention rates, and preliminary data on design effects. It is estimated that the intervention group will have a reduction of approximately 40% in the incidence rate of malnutrition at the final measurement compared to baseline. This study assumes $\alpha=0.05$, a test power ($1-\beta$) of at least 80%, an intraclass correlation coefficient (ICC) of ≤ 0.002, an average of 35 preschool children in each school, and the detection of a risk ratio of 0.60 with a baseline malnutrition incidence rate of 25% [7]. Therefore, a minimum of 16 schools is required. Additionally, considering the possibility of sample dropout in some schools and the potential for including fewer samples in certain schools, a total of 17 schools were recruited.

10. MEDICAL RADIATION SUBCOMMITTEE/INSTITUTION BIOLOGIC COMMITTEE APPROVAL

Not applicable.

11. IND/IDE NUMBER; INVESTIGATIONAL DRUG DATA SHEET

Not applicable.

References

1. The Lancet Child & Adolescent Health. Child malnutrition: Hungry for action. *Lancet Child Adolesc. Health* **2021**, *5*, 459. https://doi.org/10.1016/s2352-4642(21)00170-x.
2. Herrera-Cuenca, M. Understanding children's undernutrition in Venezuela. *eBioMedicine* **2021**, *67*, 103367.
3. Chabite, I.; Garrine, C.; Ferrão, L.; Fernandes, T. Malnutrition and school feeding programmes. *J. Nutr. Heal. Food Eng.* **2018**, *8*, 340–344. https://doi.org/10.15406/jnhfe.2018.08.00292.
4. Hanel, T.; Miller, B.; Aboul-Enein, B.; Benajiba, N.; Kruk, J. Farm-to-school nutrition programs with special reference to Egypt and Morocco. *North Afr. J. Food Nutr. Res.* **2021**, *5*, 100–104. https://doi.org/10.51745/najfnr.5.12.100–104.
5. World Food Programme. Evaluation of China WFP Country Strategic Plan 2017–2021. 2021. Available online: https://www.wfp.org/publications/evaluation-china-wfp-country-strategic-plan-2017-2021 (accessed on 3 June 2024).
6. Hu, B.; Tang, S.; Wang, Z.; Chen, Y.; Chen, X.; Zhao, Q.; Jiang, Y.; Shen, M.; Zhang, C.; Kaliszewski, C.; et al. Dietary diversity is associated with nutrient adequacy, blood biomarkers and anthropometric status among preschool children in poor ethnic minority area of Northwest China. *Front. Nutr.* **2022**, *9*, 948555. https://doi.org/10.3389/fnut.2022.948555.
7. China, Impact Evaluation of the Preschool Nutrition Pilot in Selected Counties of Xiangxi Prefecture, Hunan, PR China. 2022. Available online: https://www.wfp.org/publications/china-preschool-nutrition-pilot-selected-countries-china-feb-2018-jan-2021-evaluation-baseli (accessed on 3 June 2024).
