# Supplementary material for: Effect of Customized Nutritious Breakfast and Nutrition Education on Nutritional Status of Preschool Children in Economically Underdeveloped Multi-Ethnic Areas: A Cluster Randomized Clinical Trial in Linxia, China
Source: Nutrients. 2024 Jul 16;16(14):2287. doi: 10.3390/nu16142287 (PMC11280342; doi:10.3390/nu16142287)

The breakfast recipe for each grade

1. Determination of dietary energy target for the sample children

(1) Energy requirement of children in junior class: group energy requirement target = Σ different (subgroup energy rni × number of people/group) ÷ total number of people = (1250 × 423 + 1200 × 342) ÷ 765 = 1227kcal

(2) Energy requirement of children in middle class: 1300kcal

(3) Energy requirement of children in older classes: 1400kcal

2. Macronutrient dietary targeting for sample children

Proportion of macronutrients supplied to children: 14% protein, 30% fat, 56% carbohydrates

(1) Dietary protein intake target for junior class (g) = 1227kcal × 14% ÷ 4kcal/g = 43g

Dietary fat intake target for junior class (g) = 1227kcal × 30% ÷ 9kcal/g = 41g

Dietary carbohydrate intake target for junior class (g) = 1227kcal×56%÷4kcal/g = 172g

(2) Dietary protein intake target for children in middle school (g) = 1300kcal×14%÷4kcal/g = 45.5g

Dietary fat intake target for middle school children (g) = 1300kcal×30%÷9kcal/g = 43.3g

Dietary Carbohydrate Intake Targets for Middle-aged Children (g) = 1300kcal×56%÷4kcal/g = 182g

(3) Dietary protein intake target for senior children (g) = 1400kcal × 14% ÷ 4kcal/g = 49g

Dietary fat intake target for senior children (g) = 1400kcal×30%÷9kcal/g = 46.7g

Dietary carbohydrate intake target (g) = 1400kcal×56%÷4kcal/g = 196g

3. Calculation of Reference Intake of Nutrients for Breakfast for Sample Children

Breakfast should account for 30% of total energy, so breakfast nutrient intake target:

(1) Junior Class of kindergarten:

Energy (kcal) = 1227kcal×30% = 368kcal

Protein intake (g) = 43g×30% = 12.9g

Fat intake (g) = 41g×30% = 12.3g

Carbohydrate intake (g) = 172g x 30% = 51.6g

(2) Middle Class of kindergarten:

Energy (kcal) = 1300kcal x 30% = 390kcal

Protein intake (g) = 45.5g×30% = 13.7g

Fat intake (g) = 43.3g x 30% = 13.0g

Carbohydrate intake (g) = 182g x 30% = 54.6g

(3) Senior Class of kindergarten:

Energy (kcal) = 1400kcal x 30% = 420kcal

Protein intake (g) = 49g×30% = 14.7g

Fat intake (g) = 46.7g×30% = 14.0g

Carbohydrate intake (g) = 196g x 30% = 58.8g

4. Two-week Recipes

According to the target intake of each nutrient, various types of food were determined in the order of staple food → side dishes → vegetables → oil and salt, and the recipes are prepared as follows:

Recipe 1:

	Monday		Tuesday		Wednesday		Thursday		Friday	
	Dishs	Ingredients	Dishs	Ingredients	Dishs	Ingredients	Dishs	Ingredients	Dishs	Ingredients
Breakfast	Vegetable Congee	Round-grained rice 25g<br>Vegetables 20g	Purple Potato Buns	Purple Potato 15g<br>Tailared flour(grade one) 40g	Mutton Bun (Mutton Soup)	Lamb 30g<br>Vermicelli 10g<br>Seaweed 10g<br>Cilantro 10g<br>White radish 20g<br>Tofu 30g<br>Vegetable oil 5ml	Leek and Egg Buns	Special One Flour 60g<br>Chives 30g<br>Egg 33g<br>Vegetable oil 3ml	Mixed congee	Job's Tears 10g<br>Red Bean 10g<br>Mung Bean 10g<br>Chinese wolfberry 3g<br>Dried jujube 5g<br>Lily 5g
	Steamed Buns	Tailared flour(grade one) 50g	Tomato Scrambled Eggs	Tomato 50g<br>Egg 33g<br>Vegetable oil 5ml			Milk	Milk 100ml	Fried vegetable	Seasonal vegetables (oleander, lettuce, bok choy, rapeseed, etc.)<br>Totaling 50g<br>Vegetable oil 6ml
	Egg custard	Egg 33g<br>Sesame oil 5ml	Oatmeal Milk	Oats 20g<br>Milk 100ml	Steamed Buns	Tailared flour (grade one) 50g	Large almonds	Large almonds (without shell) 5g	Egg Mantou	Egg 10g<br>Tailared flour(grade one) 30g
	Yogurt	Yogurt 94g							Yogurt	Yogurt 90g
	Apple	Apple 65g								

Recipe 2*:

	Monday		Tuesday		Wednesday		Thursday		Friday	
	Dishs	Ingredients	Dishs	Ingredients	Dishs	Dishs	Ingredients	Dishs	Ingredients	Dishs
Breakfast	Hezhou Baozi	Tailared flour (grade one) 55g<br>Kale 50g<br>Lamb 10g<br>Vegetable oil 4ml	Chicken Congee with Shiitake Mushroom	Shiitake mushroom 10g<br>Chicken 10g<br>Round-grained rice 15g	Carrot and Sesame Buns	Tailared flour (grade one) 45g<br>Carrot 5g<br>Sesame 5g	Beef Steamed Bun (Beef Soup)	Beef 30g<br>Vermicelli 10g<br>Seaweed 10g<br>Cilantro 10g<br>White radish 20g<br>Vegetable oil 5ml	Pumpkin Porridge	Millet 25g<br>Pumpkin 30g
	Milk	Milk 100ml	Red Bean Bun	Tailared flour (grade one) 30g<br>Red bean paste 30g	Egg custard	Egg 33g<br>Sesame oil 5ml	Steamed Buns	Tailared flour (grade one) 50g	Egg custard	Egg 33g<br>Sesame oil 5ml
	Boiled egg	Egg 33g	Boiled quail eggs	Quail eggs 35g	Yogurt	Yogurt 90g	Yogurt	Yogurt 90g	Steamed Broccoli	Broccoli 50g<br>Vegetable oil 3ml
			Walnut	Walnut kernel 10g	Apple	Apple 65g			Yogurt	Yogurt 90g
									Steamed Buns	Tailared flour (grade one) 50g

Note: Amount of ingredients prepared = amount of ingredients x total number of children in kindergarten.

5. Recipe illustrations

Monday

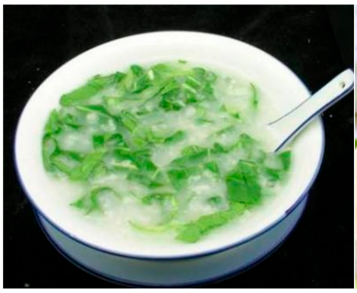

Vegetable Congee

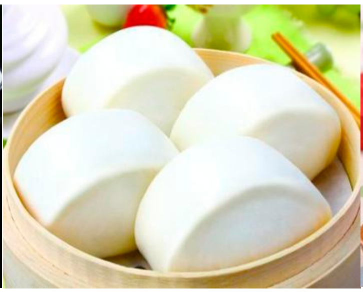

Steamed Buns

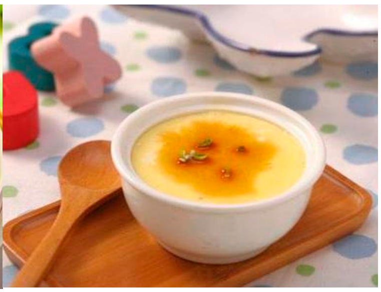

Egg custard

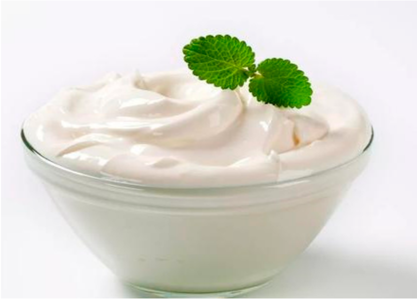

Yogurt

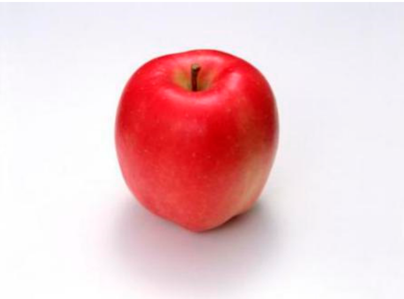

Apple

Tuesday

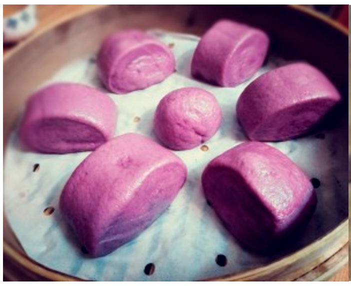

Purple Potato Buns

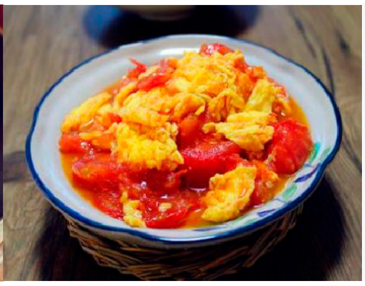

Tomato Scrambled Eggs

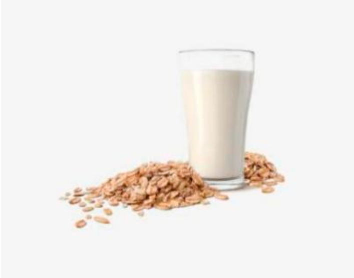

Oatmeal Milk

Wednesday

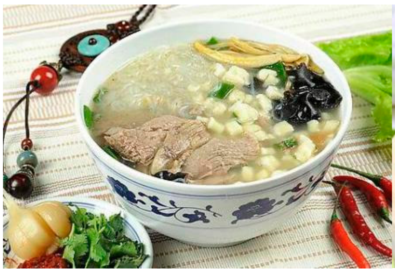

Mutton Bun (Mutton Soup)

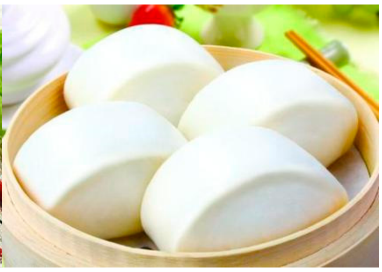

Steamed Buns

Thursday

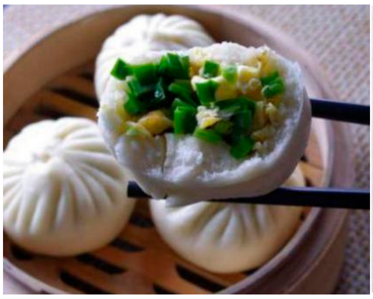
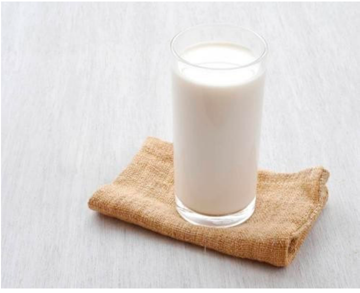
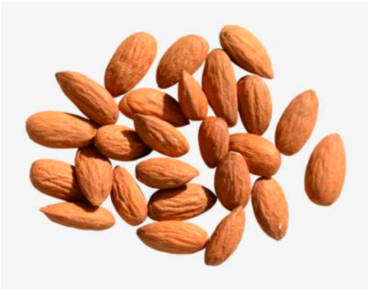

Leek and Egg Buns Milk Large almonds

Friday

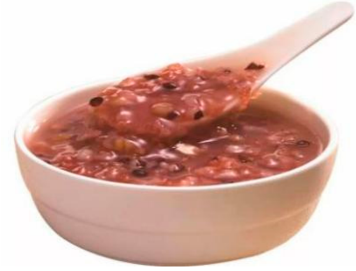
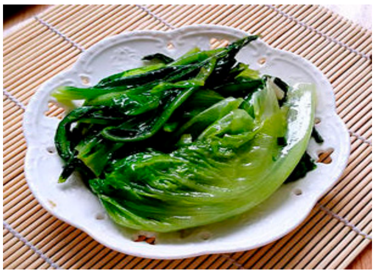

Mixed congee Fried vegetable

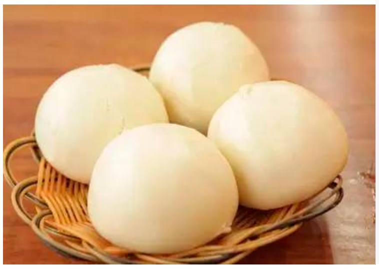
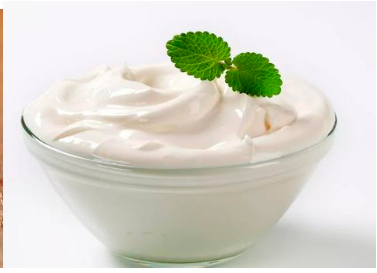

Egg Mantou Yogurt

Monday

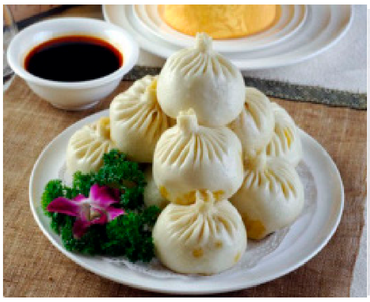
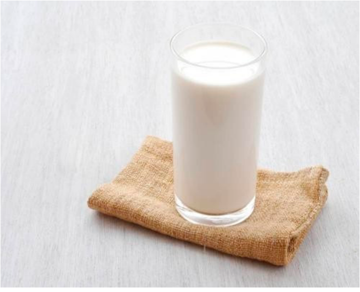
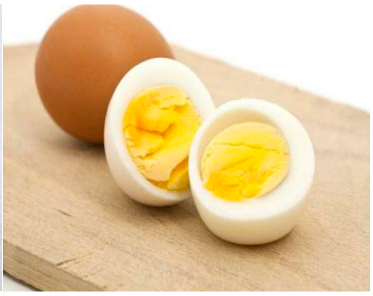

Hezhou Baozi Milk Boiled egg

Tuesday

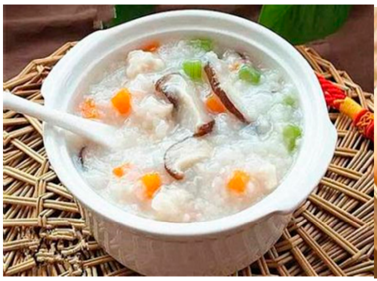
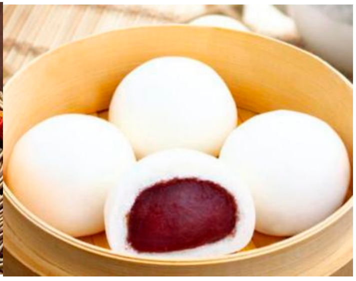

Chicken Congee with Shiitake Mushroom Red Bean Bun

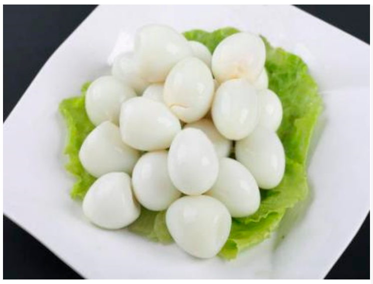
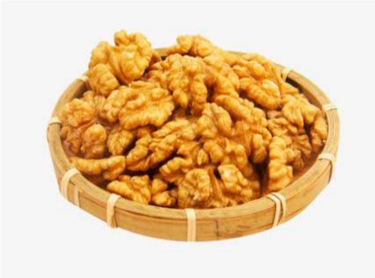

Boiled quail eggs Walnut

Wednesday

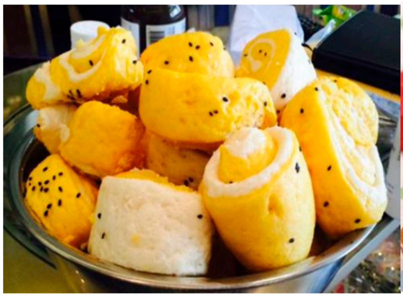
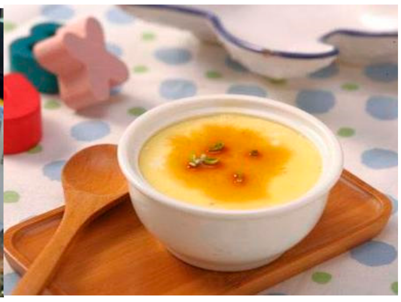

Carrot and Sesame Buns Egg custard

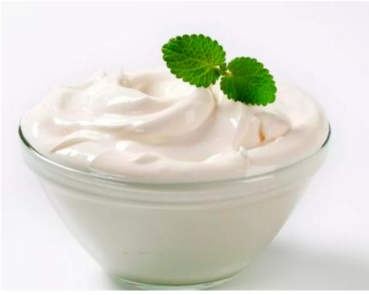 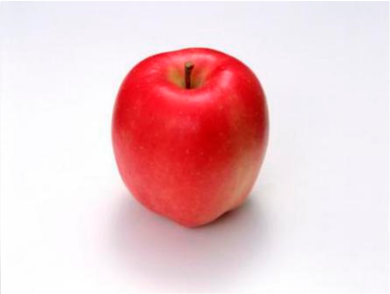

Yogurt Apple

Thursday

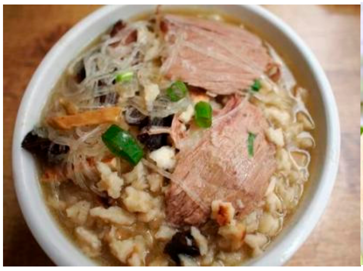 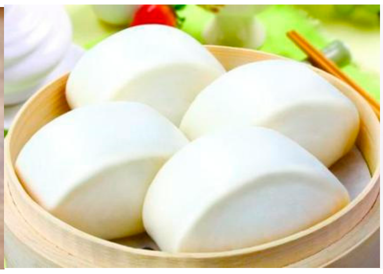 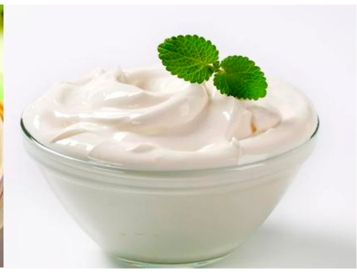

Beef Steamed Bun (Beef Soup) Steamed Buns Yogurt

Friday

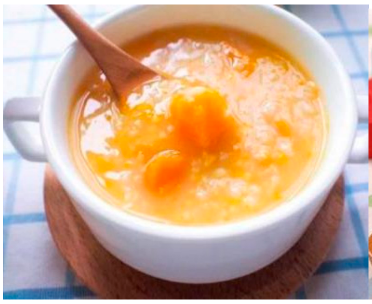 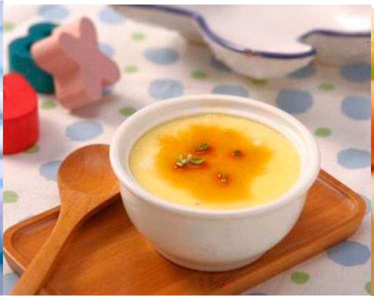 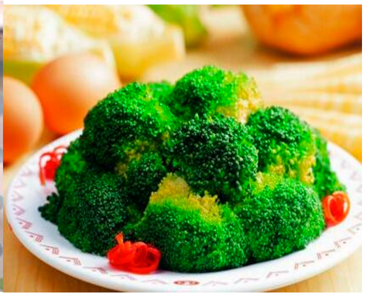

Pumpkin Porridge Egg custard Steamed Broccoli

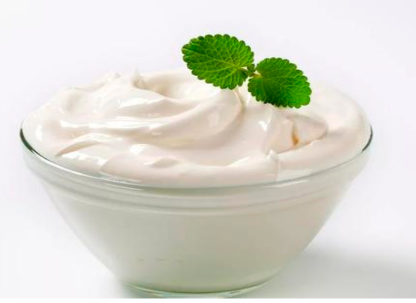 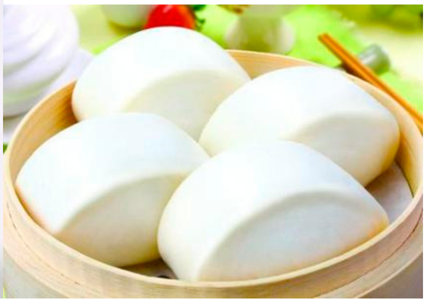

Yogurt Steamed Buns

6. Provision of local ingredients

6.1 Ingredients with high calcium content

Milk, shrimp skin, sesame paste, alfalfa, kelp, peanut kernels, nori, black fungus, etc.

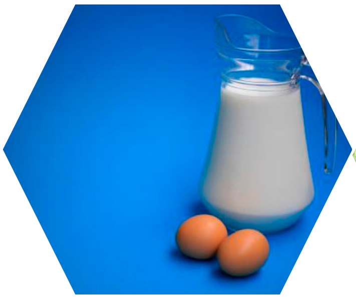
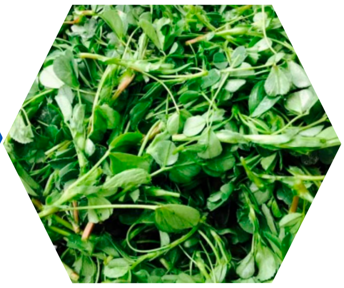

6.2 Ingredients with high iron content

Buckwheat (with skin), black fungus (dried), nori (dried), animal liver, tofu skin, sesame paste, mushrooms (dried), blood products, and lentils.

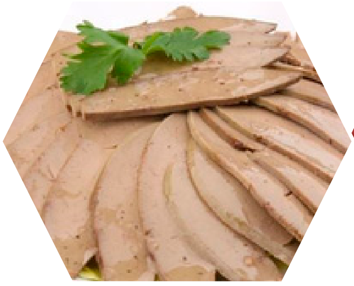
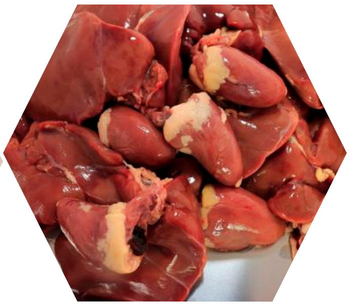

6.3 Ingredients with high zinc content

Wheat germ flour, walnuts, lamb, beef, animal offal, eggs, beans, oats and peanuts.

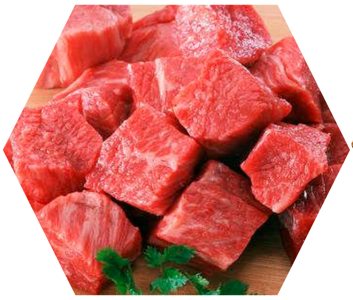
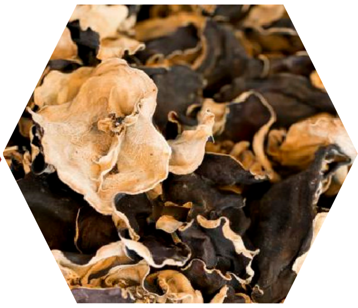

Supplement: Supplementary file 1 [file nutrients-16-02287-s001.zip › Supplementary File S2.pdf]
