# Supplementary material for: Effect of Customized Nutritious Breakfast and Nutrition Education on Nutritional Status of Preschool Children in Economically Underdeveloped Multi-Ethnic Areas: A Cluster Randomized Clinical Trial in Linxia, China
Source: Nutrients. 2024 Jul 16;16(14):2287. doi: 10.3390/nu16142287 (PMC11280342; doi:10.3390/nu16142287)
Supplement: Supplementary file 1 [file nutrients-16-02287-s001.zip › Supplementary Tables.pdf]

Supplement table S1. Demographic differences between drop-out and retention samples

Category	Total	Drop-out sample	Retention sample	*P* Value
	N=578	n=128	n=450	
BMI	14.50±1.15	14.47±1.16	14.51±1.15	0.748
BMIz	0.86±0.83	0.84±0.83	0.86±0.82	0.819
Age	3.57±0.56	3.62±0.60	3.52±0.52	0.042
Gender				0.470
Male	321(55.54%)	67(52.34%)	254(56.44%)	
Female	257(44.46%)	61(47.66%)	196(43.56%)	
Ethnicity				0.001
non-Muslim	323(56.90%)	46(35.94%)	277(61.56%)	
Muslim	255(44.10%)	72(56.25%)	183(40.67%)	
Developmental status				
Stunting	39(6.75%)	9(7.03%)	30(6.67%)	1.000
Underweight	40(6.92%)	10(7.81%)	30(6.67%)	0.843
Wasting	24(4.15%)	3(2.34%)	21(4.67%)	0.343

Supplement table S2. Tests for two proportions in a cluster-randomized design

Power	Group 1 Clusters/Items K1/M1	Group 2 Clusters/Items K2/M2	Group 1 Prop P1	Group 2 Prop P	Diff D1	IntraCluster Corr. (ICC)	Alpha
0.823	8/35	8/35	0.25	0.15	0.1	0.002	0.05

Test Statistic:7-Test (Unpooled),H0: P1 - P2 =0 vs. H1: P1 - P20Hypotheses
